# Effect of aging on muscle and tendon properties in highly functioning elderly people

**DOI:** 10.1111/sms.13372

**Published:** 2019-04-29

**Authors:** Birgit Pötzelsberger, Alexander Kösters, Thomas Finkenzeller, Erich Müller

**Affiliations:** ^1^ Department of Sport Science and Kinesiology University of Salzburg Salzburg Austria

**Keywords:** active lifestyle, habituation, longitudinal study, muscle, tendon

## Abstract

This study analyzes long‐term changes in muscle strength, muscle architecture, and patellar tendon mechanical properties in a specific sample of physically active elderly people. Twenty‐two participants were re‐examined from a former 12‐week‐long skiing intervention study: 11 from the intervention group (IG: 7 ♀, 4 ♂; 67 ± 3 years) and 11 from the control group (CG: 6 ♀, 5 ♂; 66 ± 4 years). Muscle architecture, strength endurance, maximum torque, and tendon properties were analyzed three times within 6 months, and again 6 years later in a follow‐up test. No changes in either group could be observed between June 2009 and April 2015 in any parameter. This can be interpreted positively because no age‐related decreases were found. Although our participants were physically active from the very beginning (>150 min/wk), it must be noted that the intensity of the physical activity was too low to provoke physiological improvements in leg strength or muscle/tendon morphology.

## 1 INTRODUCTION

Current demographic trends show that life expectancy is increasing worldwide. The aging process in humans is accompanied by inherent physiological changes, including a decrease in muscle strength.^1^, ^2^, ^3^, ^4^, ^5^ Senile sarcopenia, the loss of muscle mass in older people, and changes in muscle architecture and tendon properties, as well as reduced neural drive, are known to account for a diminished mechanical output of the muscle‐tendon unit in the elderly.^6^

Physical activity is a possible tool to counteract these changes during the aging process. Muscular adaptations can be achieved even in the elderly due to mechanical stimulation of the musculo‐skeletal system, which causes changes in muscle morphology, muscle architecture, and strength output.^7^, ^8^ Regarding the tendon, a recent review of Svensson et al^9^ indicated major changes in mechanical and material properties of tendon tissue during maturation rather than aging. During aging, it is assumed that a reduction in tendon properties, seen as a decrease in modulus and stiffness, contributes to an increased risk of injuries. Physical activity appears to be instrumental in the mitigation of aging‐related declines.^9^

Loss of muscle strength is an accurate predictor of a functional limitation associated with increased risk of falls, loss of physical independence, and quality of life.^10^, ^11^, ^12^ The importance of physical activity is well accepted to counter these physiological declines (muscle strength and aerobic endurance).^13^, ^14^ Guidelines recommend at least 150 minutes of moderate intensity, or at least 60 minutes of vigorous‐intensity aerobic physical activity weekly, or an equivalent combination thereof, as well as 2 days of strengthening activities per week to protect against the age‐associated loss of muscle mass or physical performance.^15^ A substantial number of older adults do not meet these physical activity recommendations to promote their health, and furthermore, it has been shown that with increasing age, they become more sedentary than younger adults.^16^

Regarding physical activity, retirement is a major transition in the course of life. The effect of the transition to retirement on exercise habits is equivocal.^17^ On the one hand, a person may experience a decline of physical activities performed during work or while commuting to and from work, as a result of retirement. On the other hand, it may also reduce time‐related barriers to sports and non‐sports leisure‐time physical activities. Singerland et al^18^ reported a relative reduction in physical activity through the transition to retirement. If that is the case, then, there is a need to promote physical activity in this sensitive stage of life. Alpine skiing is a very popular leisure‐time physical activity in the alpine regions and seems to be suitable for keeping activity level high during winter months. Previous investigations have shown that alpine skiing could provide an appropriate health‐enhancing activity for the elderly.^19^, ^20^ High variations in snow conditions, slope inclination and preparation, speed and turning radius, as well as the dominant eccentric quadriceps muscle activity required throughout the turning cycle, might counteract the age‐related degeneration process and loss of muscle function.^21^

Within the framework of the 12‐week intervention study (SASES: Salzburg Skiing for the Elderly Study), it was shown that alpine skiing is an effective intervention to elicit adaptive changes in leg muscle mass, strength, and patellar tendon mechanical and material properties.^8^, ^22^, ^23^

All participants involved in the SASES were highly active. Therefore, it seems important to verify whether they could maintain their high level of physical activity with increasing age. The aim of the study was as follows: (a) to investigate whether a 12‐week ski intervention was sufficient to instill long‐term moderate‐intensity exercise habits in everyday life, and (b) whether participants benefit from the positive effects with regard to muscle architecture, muscle strength, and tendon properties shown in the SASES.

## 2 METHODS

### 2.1 Participants

The participants from the SASES were invited to participate in the present follow‐up study. Of the 42 potential candidates (IG: 22, CG: 20), 25 participants agreed to take part. Out of these, three participants were excluded due to stroke (n = 1), heart disease (n = 1), and vacation at t3 (n = 1). A total of 22 participants were involved in the follow‐up study, 11 from the intervention group (IG: 7 ♀, 4 ♂; 67 ± 3 years) and 11 from the control group (CG: 6 ♀, 5 ♂; 66 ± 4 years). Reasons for nonparticipating in the follow‐up study ranging from not being able to participate at the fixed measurement time due to vacation or other reasons (n = 3), no motivation to participate again (n = 7), lost to follow‐up (n = 6), and due to injury (n = 1).

### 2.2 Physical activity

The participants received a comprehensive questionnaire to fill out within 1 week at home (paper‐pencil test). Physical activity was assessed by asking the duration and frequency of physical leisure time and sport activities per week (free answer possibility) in accordance with Stiller.^24^ The total time of physical activity was computed by multiplying the duration and the frequency of each activity. Subsequently, the values were totaled. In contrast to t1, t2, and t3, at the follow‐up test (t4), the participants were asked relating to the time span of the last 5 years. Detailed description of the comprehensive questionnaire is provided in the article on the study design within this supplement.^25^

### 2.3 Test procedure

Muscle architecture and strength endurance tests were conducted on the first test day, and maximum torque and the measurement of tendon properties on the second test day. The assessments were separated by 48‐72 hours of rest.

#### 2.3.1 Test day 1

*Muscle architecture* Pennation angle (*θ*, °) and fascicle length (*L*_f_, mm) of the vastus lateralis (VL) muscle were determined in vivo at rest. Measurements were performed with the subject lying supine on an examination couch, using a digital ultrasonographer (MyLab25; Esaote, Genoa, Italy). Details of the measurement procedure and data analysis are presented in Narici et al.^8^

*Warm‐up* Prior to strength and tendon measurements, participants completed a general warm‐up consisting of 10 minutes of supervised cycling on a stationary ergometer (Heinz Kettler GmbH and Co. KG, Ense‐Parsit, Germany) at a submaximal workload of 0.75 W/kg body mass with a cadence of ~70 revolutions/min.

*Strength endurance* was measured isokinetically using the manufacturer's leg press module (IsoMed 2000; D&R Ferstl GmbH, Hemau, Germany). The participants wore standardized testing shoes without any additional damping elements during this test. They were seated on the dynamometer chair in an upright position (backrest 105°; 180° corresponding to full extension). The shoulders and pelvis were fixed to minimize extraneous body movement during testing. Handgrips were used during testing for further stabilization of the upper body. After fixation of the upper body, the range of motion was set between 100° and 150° of knee flexion using a handheld goniometer. Once in position, participants submaximal extended their legs against the force plate. For testing, a translational velocity of 100 mm/s was used. Prior to testing, 10 submaximal familiarization trials were performed. After 1 minute of rest, participants had to perform 35 maximum concentric leg extensions. Between single contractions, there was a rest period in which the device passively flexed the participant's legs back to the starting position (=100°). Therefore, the same velocity was used and the loading and relief phases were of equal duration for each subject (ie, 1‐2 seconds, depending on the participants’ lower extremities anthropometric characteristics). The test instruction was to push as fast and forcefully as possible throughout the complete range of motion. The average work (AW) and average peak force (APF) calculated throughout all testing trials for bilateral legs, as well as for unilateral legs served as the outcome parameters for strength endurance performance.^22^

#### 2.3.2 Test day 2

*Maximum torque* of the knee extensor and flexor muscles was measured isometrically (IsoMed 2000; D&R Ferstl GmbH), only for the right leg and using the dynamometer in combination with a unilateral knee attachment. After fixation of the upper body as described above, the mechanical axis of the dynamometer was aligned with the knee's axis of rotation. The shin pad of the dynamometer lever arm was fastened approximately 3 cm superior to the lateral malleolus. Gravity adjustment was performed using the integrated software. The test procedure was divided into two test series, namely knee extension and flexion, both at a knee angle of 90°. Each test series included a submaximal familiarization trial followed by three maximal test trials (20‐seconds rest interval between single trials, 3‐minutes break between series). The test instruction was to push as forcefully as possible. The highest torque generated during the three maximal voluntary contractions (MVC) was taken for data analysis. Throughout the testing protocol, strong verbal encouragement and visual online feedback were provided to each participant.^22^

*Tendon size and mechanical properties* Tendon length and cross‐sectional area (CSA) were measured from ultrasound scans (10‐15 MHz transducer; MyLab25; Esaote) in the longitudinal and transversal planes. Patellar tendon length was measured externally as the distance between the tibial enthesis and apex of the patella. In the transversal plane, CSA was measured along the tendon, below the apex of the patella (CSAd), in the mid portion (CSAm), and just above the distal insertion (CSAp).

Tendon stiffness and Young's modulus were measured in vivo, by quantifying the elongation of the tendon during a ramp isometric contraction and measuring the slope of the force‐elongation curve in the highest force region. Measuring procedure and data analysis can be seen in detail by Seynnes et al.^23^

### 2.4 Statistical analysis

For descriptive statistics, data are presented as means and SD. A 4 × 2 ANOVA with repeated measures (time [t1, t2, t3, t4] × group [IG, CG]) was calculated. Greenhouse‐Geisser values are reported in the case of the violation of sphericity. In case of significant interaction effects, univariate tests using Bonferroni adjustment were conducted, to verify the differences within each group between the different time points. The level of significance was set at *α* < 0.05. All statistical analyses were performed using the Statistical Package for Social Sciences (SPSS; Version 23 for windows; SPSS Inc., Chicago, IL, USA).

## 3 RESULTS

The self‐reported physical activity in minutes per week remained unchanged in both groups over time (Tables 1 and 2).

**Table 1:** Descriptive statistics (mean ± SD) of physical activity, muscle strength, and CSA of the patellar tendon

Measure	*n*_1_/*n*_2_	December 2008/January 2009 (t1)		April 2009 (t2)		June 2009 (t3)		April 2015 (t4)	
		IG	CG	IG	CG	IG	CG	IG	CG
Physical activity (min/wk)	11/10	722 ± 191	555 ± 348	574 ± 248	612 ± 393	653 ± 279	605 ± 294	567 ± 305	454 ± 279
*M*_max_ flexion (Nm/kg)	9/11	0.89 ± 0.20	0.97 ± 0.16	0.99 ± 0.21	1.04 ± 0.24	0.90 ± 0.23	0.95 ± 0.16	0.95 ± 0.22	0.96 ± 0.20
*M*_max_ extension (Nm/kg)	11/11	1.96 ± 0.48	1.86 ± 0.40	1.89 ± 0.51	1.89 ± 0.45	1.99 ± 0.59	1.89 ± 0.44	1.89 ± 0.57	1.81 ± 0.54
APF (left leg) (N/kg)	8/10	12.69 ± 2.83	11.74 ± 3.37	14.79 ± 2.82	14.19 ± 3.34	15.41 ± 3.61	13.98 ± 3.08	14.97 ± 3.15	13.32 ± 2.37
APF (right leg) (N/kg)	8/10	12.44 ± 2.69	11.76 ± 3.82	15.13 ± 2.59	14.47 ± 3.69	15.63 ± 3.10	13.94 ± 3.46	14.70 ± 2.90	13.54 ± 2.80
AW (left leg) (J/kg)	9/10	0.73 ± 0.22	0.73 ± 0.21	0.82 ± 0.22	0.88 ± 0.21	0.79 ± 0.36	0.82 ± 0.34	0.83 ± 0.22	0.84 ± 0.19
AW (right leg) (J/kg)	9/10	0.72 ± 0.20	0.74 ± 0.22	0.84 ± 0.18	0.91 ± 0.22	0.79 ± 0.34	0.90 ± 0.22	0.81 ± 0.20	0.87 ± 0.20
PT CSA p (mm²)	10/9	82.73 ± 12.85	87.20 ± 19.38	86.47 ± 13.59	88.90 ± 21.23	83.50 ± 12.62	89.06 ± 19.04	86.34 ± 12.02	86.93 ± 12.29
PT CSA m (mm²)	10/9	89.64 ± 14.08	94.84 ± 16.98	89.13 ± 14.09	94.36 ± 19.90	88.80 ± 14.50	94.47 ± 18.56	92.93 ± 14.31	96.93 ± 18.54
PT CSA d (mm²)	10/9	103.82 ± 23.96	107.19 ± 21.03	105.62 ± 22.21	104.89 ± 20.89	105.00 ± 21.53	104.05 ± 20.16	106.66 ± 12.34	109.22 ± 19.70

*Note*.

APF, average peak force; AW, average work; PT CSA p, m, d = patellar tendon cross‐sectional area proximal, medial, distal; *n*_1_ = sample size of the intervention group (IG); *n*_2_ = sample size of the control group (CG); *M*_max_ = maximum torque.

**Table 2:** Results of Time × Group ANOVA with repeated measures of physical activity, muscle strength, and CSA of the patellar tendon

	Time				Group				Time × Group			
	*F*	*P*	*η*²	1−*β*	*F*	*P*	*η*²	1−*β*	*F*	*P*	*η*²	1−*β*
Physical activity (min/wk)	*F*_3.57_ = 1.80	0.158	0.09	0.44	*F*_1.19_ = 0.48	0.495	0.03	0.10	*F*_3.57_ = 1.04	0.376	0.05	0.27
*M*_max_ flexion (Nm/kg)	*F*_3.54_ = 3.40	<0.05*	0.16	0.74	*F*_1.18_ = 0.29	0.596	0.02	0.08	*F*_3.54_ = 0.49	0.690	0.03	0.14
*M*_max_ extension (Nm/kg)	*F*_3.60_ = 1.11	0.335	0.05	0.22	*F*_1.20_ = 0.13	0.723	0.01	0.06	*F*_3.60_ = 0.44	0.624	0.02	0.11
APF (left leg) (N/kg)	*F*_3.48_ = 17.07	<0.001***	0.52	1.00	*F*_1.16_ = 0.70	0.416	0.04	0.12	*F*_3.48_ = 0.75	0.530	0.04	0.20
APF (right leg) (N/kg)	*F*_3.48_ = 19.58	<0.001***	0.55	1.00	*F*_1.16_ = 0.54	0.475	0.03	0.11	*F*_3.48_ = 0.71	0.549	0.04	0.19
AW (left leg) (J/kg)	*F*_3.54_ = 4.40	<0.05*	0.20	0.61	*F*_1.18_ = 0.05	0.829	0.00	0.06	*F*_3.54_ = 0.29	0.680	0.02	0.09
AW (right leg) (J/kg)	*F*_3.51_ = 6.58	<0.01**	0.28	0.83	*F*_1.17_ = 0.49	0.495	0.03	0.10	*F*_3.51_ = 0.56	0.542	0.03	0.13
APF (bilateral) (N/kg)	*F*_3.51_ = 16.86	<0.001***	0.50	1.00	*F*_1.17_ = 0.91	0.354	0.05	0.15	*F*_3.51_ = 0.28	0.843	0.02	0.10
AW (bilateral) (J/kg)	*F*_3.51_ = 25.01	<0.001***	0.60	1.00	*F*_1.17_ = 0.02	0.890	0.00	0.05	*F*_3.51_ = 0.41	0.746	0.02	0.13
PT CSA p (mm²)	*F*_3.51_ = 0.84	0.423	0.05	0.17	*F*_1.17_ = 0.23	0.640	0.01	0.07	*F*_3.51_ = 0.80	0.435	0.05	0.16
PT CSA m (mm²)	*F*_3.51_ = 3.34	<0.05*	0.16	0.73	*F*_1.17_ = 0.46	0.506	0.03	0.10	*F*_3.51_ = 0.18	0.911	0.01	0.08
PT CSA d (mm²)	*F*_3.51_ = 1.04	0.343	0.06	0.18	*F*_1.17_ = 0.01	0.908	0.00	0.05	*F*_3.51_ = 0.59	0.504	0.03	0.12
PT stiff (N/mm)	*F*_3.48_ = 1.30	0.284	0.07	0.33	*F*_1.16_ = 3.15	0.095	0.17	0.39	*F*_3.48_ = 3.45	<0.05*	0.18	0.74
PT YM (Gpa)	*F*_3.48_ = 1.11	0.354	0.07	0.28	*F*_1.16_ = 4.74	<0.05*	0.23	0.53	*F*_3.48_ = 3.02	<0.05*	0.16	0.68
Pennation angle (°)	*F*_3.54_ = 0.99	0.407	0.05	0.25	*F*_1.18_ =0.82	0.377	0.04	0.14	*F*_3.54_ = 4.71	<0.01**	0.21	0.87
Fascicle length (cm)	*F*_3.54_ = 1.14	0.343	0.06	0.29	*F*_1.18_ = 2.60	0.124	0.13	0.33	*F*_3.54_ = 1.51	0.223	0.08	0.38

*Note*.

APF, average peak force; AW, average work; *M*_max_ = maximum torque; PT CSA p, m, d = patellar tendon cross‐sectional area proximal, medial, distal.

*P < 0.05; **P < 0.01, ***P < 0.001.

**Figure 1:**
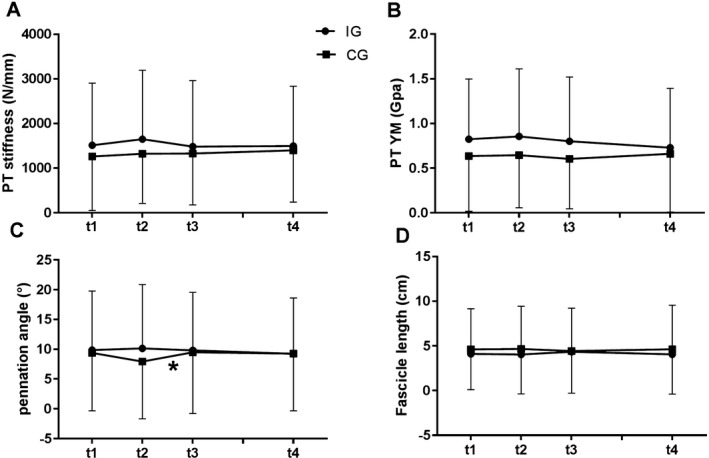
Patellar tendon stiffness (panel A), tendon Young's modulus (panel B), pennation angle (panel C), and fascicle length (panel D) at the three measurements in 2009 (before, immediately after the intervention and a retention test in June 2009) and in April 2015

Regarding muscle architecture, no time‐by‐group interaction effect and no main effect were found for the fascicle length of the muscle vastus lateralis (Figure 1D). A significant time‐by‐group interaction effect was found in the pennation angle of the muscle vastus lateralis with a significant increase between t2 and t3 in the CG (*P *< 0.05; t1: 14.74 ± 1.16° vs t2: 16.74 ± 2.23°; Figure 1C and Table 2).

**Figure 2:**
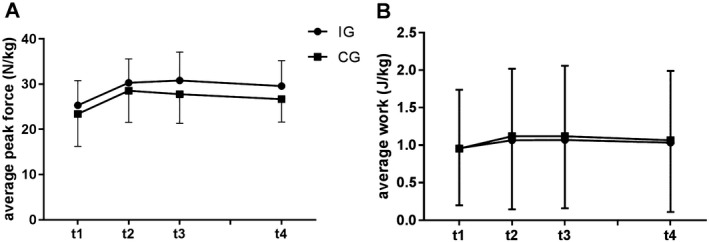
Average peak force (panel A) and average work (panel B) measured bilateral at the three measurements in 2009 (before, immediately after the intervention and a retention test in June 2009) and in April 2015

For maximum isokinetic strength endurance, no time‐by‐group interaction effects were found in the APF and AW for unilateral (Tables 1 and 2) and bilateral tests (Figure 2). However, a main effect of time was observed for the APF (unilateral *P *< 0.001; bilateral: *P *< 0.000). Between t1 and t2 and t1‐t3 increases in unilateral and bilateral APF were observed (all *P *< 0.001), whereas APF revealed an increase between t1 and t4 (unilateral: *P *< 0.01; bilateral: *P *< 0.05). A main effect of time was determined for the AW during the unilateral tests (left leg *P *< 0.05; right leg *P *< 0.01) and during the bilateral test (*P *< 0.001). An increase was observed between t1 and t2 (unilateral and bilateral *P *< 0.001), between t1 and t4 (unilateral and bilateral *P *< 0.01), and between t1 and t3 (bilateral: *P *< 0.001). These data are summarized in Tables 1 and 2 and Figure 2.

No time‐by‐group interaction effects were evident for isometric torque in knee extension or flexion. A main effect of time was observed for maximum torque during the isometric knee flexion (*P *< 0.05) with a decrease between t2 and t3 (*P *< 0.05; Tables 1 and 2).

Focused on the tendon mechanical properties, a time‐by‐group interaction effect was determined for the patellar tendon stiffness (*P *< 0.05) and the Young's modulus (*P *< 0.05). For Young's modulus, a group effect also was found (*P *< 0.05; Figure 1A,B and Table 2). Analysis revealed no further differences in each group between the different time points. For the patellar tendon CSA, no time‐by‐group interaction effect was determined for CSA posterior (CSAp), CSA medial (CSAm), and CSA distal (CSAd). A main effect of time was observed for the CSAm with no further changes (Tables 1 and 2).

## 4 DISCUSSION

The unique feature of this report is the recruitment of highly active participants from a former study and the implementation of a further identical test session. Therefore, the sample of our study allows us to evaluate changes in muscle architecture, muscle strength, and tendon properties that occurred between the time of the initial intervention study and the follow‐up, some 6 years later. Furthermore, it could also be determined whether the ski intervention affected the amount of physical activity performed by elderly people in order to obtain a deeper understanding concerning habituation effects. As already described, it is particularly important for the maintenance of the health and physiological performance in old age that individuals retain a physically active lifestyle.

### 4.1 Physical activity

Our results demonstrate that, with the exception of two participants, all subject reached the goal of physical activity above 150 min/wk as recommended by the American College of Sports Medicine.^15^ Interestingly, the self‐reported amount of physical activity did not change significantly over the 6‐year follow‐up period, which was also observed in previous studies.^16^, ^18^ However, individually, large differences were found (at t4: from 150 to 1260 min/wk for example), resulting in an inhomogeneity of the parameter physical activity, not only between participants, but also over time for each individual. Nevertheless, all subjects were highly active regardless of whether they were assigned to the intervention or the control group. Details are provided by Finkenzeller et al.^25^

In addition, it could be observed that about one‐third of the individuals, who participated in the 12‐week skiing intervention, retained their skiing habits introduced during the intervention. These individuals joined the skiing exercise together as a group, 3 d/wk, in the same manner as during the intervention 6 years ago. Similar results regarding the efficacy of intervention studies on long‐term adherence to physical activity were reported.^26^ However, in contrast to many other studies that promoted ongoing exercise participation with different possibilities such as telephone follow‐up, home exercise programs, activity monitors, or Web‐based activity diaries, we did not actively motivate our participants to continue skiing. The group organized itself independently and appears to have benefited from the new social contacts established during the intervention. The importance of social contacts in terms of adherence of an exercise program is also described in Jansons et al.^26^

### 4.2 Muscle architecture

The process of aging leads to a deterioration in muscle function that is determined by structure and morphology at the architectural level.^7^ These changes are pronounced in the quantity (muscle volume) and quality (muscle architecture) of muscle. In our study, we found no changes in muscle architecture at the 6‐year follow‐up (t3‐t4). This is an unexpected finding in contrast to previous research who found a decrease in CSA of about 40% between the ages of 20 and 80 years.^27^, ^28^ The decline in muscle mass is thought to be strongly associated with a reduction in number and size of muscle fibers. Approximately 70% the vastus lateralis CSA is composed of muscle fibers in young adults, whereas in the elderly, the remaining CSA of muscle fibers is about 50%.^29^ Fascicle length and pennation angle decrease during the aging process of muscle.^30^ In the literature, conflicting results regarding the response of resistance training on muscle architecture are discussed. Scanlon et al^7^ found no changes in fascicle length and pennation angle after a 6‐week training intervention, whereas Reeves et al^31^ found increases in these parameters after training for 14 weeks. It could be assumed that these inconsistencies are caused by different intensity and duration of the training intervention. Therefore, it seems that the intensity of the physical activity of our participants was too low to provoke muscular adaptations. Regardless of being in the intervention or the control group, it has to be pointed out that the participants of our study were able to maintain their muscle function, which might be a consequence of their sportive lifestyle.

### 4.3 Strength endurance and maximum torque

For maximum isokinetic strength endurance, the participants experienced significant improvements between t1 and all other time points throughout the 6 years, with no difference between the IG and the CG. Learning effects might have contributed to these findings. However, it remains unclear as to what extent the learning effects still exist in the follow‐up test. In isometric torque during the knee flexion, a time effect between t2 and t3 was observed with a significant decrease in both groups, showing a deficit of the long‐lasting intervention effect on the knee flexion torque.

If we consider only t3 and t4, no significant long‐term effects could be observed in the maximum isokinetic strength endurance and isometric torque, which could be seen as being beneficial, by keeping performance constant over the 6‐year period. In comparison, cross‐sectional studies report an age‐related annual decline of 1%‐2% in isometric strength for elderly people in general.^1^ However, in longitudinal observations, higher decreases are reported, showing a decline in muscle strength of 1.5%‐2.5% for the isokinetic knee extensor and flexors.^3^, ^32^ The reported range of alterations can be explained by different study populations, levels of physical activity, and test methodologies. To counteract this age‐related decrease, several studies using resistance training over 10 or 12 weeks have pointed out positive training effects. Improvements in muscle strength ranging from 17% to 37% can be reached by people from 60 to 97 years of age, depending on the study design as shown in Narici et al.^33^ Comparing the improvements that could be achieved through resistance training with our results, we should also note that the stimuli of physical activities, such as leisure activities, work‐related activities, and housework, on skeletal muscles was enough to maintain existing muscle profiles, but not high enough to result in muscle strength improvement.

### 4.4 Tendon size and mechanical properties

In contrast to age‐related changes described in the literature, there were no changes in CSA, material and mechanical properties of the patellar tendon between t3 and t4 in our study. The function of the muscle‐tendon unit is influenced by the muscle, its neuromuscular system, and mechanical as well as material properties of the tendon. Structural and mechanical changes in tendons largely take place during maturation rather than during aging.^9^ There seems to be an increase in CSA and a decrease in Young's modulus and stiffness in an aging tendon (Svensson et al^9^ for review). Physical activity is discussed as a way to counteract these alterations during aging. Activities like recreational alpine skiing have been shown to increase patellar tendon stiffness (14%) and Young's modulus (12%) in older individuals.^23^ More pronounced adaptations on the patellar tendon were shown by Reeves et al.^34^ Their strength training intervention in elderly people revealed an increase of 65% in tendon stiffness and by 69% in Young's modulus. As already mentioned, tissue adaptations seem to be largely dependent on intensity and duration of physical activity.

### 4.5 Limitations

Some methodological considerations concerning the participants and relevance to interpretation of the study should be discussed. All participants were already very active before participating in the study and did not change their physical activity over time. Undoubtedly, elderly physically active people are more prone to participate in such studies and undergo a large number of tests. Thus, it might be assumed that our participants were more healthy and fit than a random sample of elderly people. It should be taken into consideration that those with poorer health and/or severe disabilities dropped out, which may have lead to an underestimation of the real muscle strength changes in the general population.

In conclusion, it can be stated that an active physical lifestyle is beneficial for maintaining muscle strength, muscle architecture, and tendon properties. These results are functionally relevant as reduced muscle strength in the elderly has been associated with impaired functional performance and enhanced risk of falls.^10^, ^11^, ^12^ Nevertheless, for improvement of muscle function, resistance strength training seems to be required. Questionnaires on physical activity carried out in the framework of that examination indicate that our participants rarely perform specific exercise programs, but perform a variety of different leisure and sports activities (eg, gymnastic, dancing, tennis, skiing, swimming, biking, hiking, strength training).

## 5 PERSPECTIVES

It has been shown that a 6‐year follow‐up in elderly people is a time span that holds the risk of many dropouts, especially due to age‐related degenerative diseases and disorders. Therefore, it is recommended to design studies of shorter periods for the follow‐up test in this age group. Regarding personal feedback from the participants, it may be deduced that during the first years after the intervention, the participants seemed to benefit from the social interaction experienced during regular physical activity as a group.

It should further be noted that quite a few of the participants of the IG continued their recreational activity of alpine skiing. However, the participants who took part regularly on the ski days during the winter season decreased during the ensuing 6 years, but for various reasons. To make a statement regarding the effectiveness of alpine skiing as an activity, a higher number of participants in a follow‐up test would be necessary. This in turn could be achieved by closer time gap between the retention test and the follow‐up test.

## CONFLICTS OF INTEREST

The authors declare no conflicts of interest.
